# Analysis of non-pharmaceutical interventions and their impacts on COVID-19 in Kerala

**DOI:** 10.1038/s41598-021-04488-x

**Published:** 2022-01-12

**Authors:** Elizabeth Goult, Shubha Sathyendranath, Žarko Kovač, Christina Eunjin Kong, Petar Stipanović, Anas Abdulaziz, Nandini Menon, Grinson George, Trevor Platt

**Affiliations:** 1 Plymouth Marine Laboratory, Plymouth, UK; 2 Max Planck Institute for Infection Biology, Berlin, Germany; 3 University of Split, Faculty of Science, Split, Croatia; 4 CSIR National Institute of Oceanography, Kochi, India; 5 Nansen Environmental Research Centre – India, Kochi, India; 6 ICAR Central Marine Fisheries Research Institute, Kochi, India

**Keywords:** Applied mathematics, Viral infection

## Abstract

In the absence of an effective vaccine or drug therapy, non-pharmaceutical interventions are the only option for control of the outbreak of the coronavirus disease 2019, a pandemic with global implications. Each of the over 200 countries affected has followed its own path in dealing with the crisis, making it difficult to evaluate the effectiveness of measures implemented, either individually, or collectively. In this paper we analyse the case of the south Indian state of Kerala, which received much attention in the international media for its actions in containing the spread of the disease in the early months of the pandemic, but later succumbed to a second wave. We use a model to study the trajectory of the disease in the state during the first four months of the outbreak. We then use the model for a retrospective analysis of measures taken to combat the spread of the disease, to evaluate their impact. Because of the differences in the trajectory of the outbreak in Kerala, we argue that it is a model worthy of a place in the discussion on how the world might best handle this and other, future, pandemics.

## Introduction

The emergence of a novel coronavirus, severe acute respiratory syndrome coronavirus 2 (SARS-CoV-2)^1^, has led to a global health emergency^2^, with the resulting disease coronavirus disease 2019 (COVID-19) spreading globally. COVID-19 can manifest with no symptoms or with severe illness^3,4^, leading, in extreme cases, to death. The disease is particularly dangerous to those with underlying medical conditions and older people^3^. Non-Pharmaceutical Interventions (NPIs) have been the only tool available so far to control the virus spread^5,6^.

India has been hit hard by COVID-19, reporting over 821,000 cases by 10th July 2020^7^ despite not having reached the peak of the outbreak^8^. In the South Indian state of Kerala, the pattern has been different from the rest of India. The state has a population of 33.3 million^9^, but reported only 1,208 cases of COVID-19 as of 30th May 2020^1^, of which the majority were linked to exposed or infected people travelling into Kerala from the rest of India or abroad. Countries of comparable sizes such as Canada (population of 35.2 million^10^) reported 91,600 cases on the same day, with similar introduction date^1^. The aggressive implementation of NPIs in Kerala, including track and trace, quarantining, and lockdown, has been reported as the predominant reason for Kerala’s early success in avoiding a worse outbreak^11^.

In this paper we use a susceptible-exposed-infected-recovered (SEIR) model to evaluate, retroactively, the impact of the actions taken in Kerala to contain the disease, in the four months since the first recorded appearance of the disease in Kerala on 30th January 2020, and discuss implications for the future, in the light of the model results.

Kerala followed a multi-strand approach to contain the spread of COVID-19, from 30th January 2020 when a medical student returning from Wuhan (China) tested positive. The strategy was implemented in three stages:

- Phase 1: initial stage (January 30 to March 24);
- Phase 2: lock-down stage (March 24 to June 1); and
- Phase 3: unlock stage (June 1 onwards).

During phase 1, all travellers coming from COVID-19 case reported countries were tested and monitored from entry of the state. Furthermore, the suspected cases were placed under quarantine for 14 days in the government hospitals, and all identified primary contacts were placed under self-quarantine with strict restriction.

At the beginning of Phase 2, there were 109 confirmed cases. During Phase 2, on 16th April the national government announced the identification of hotspot districts in every state in India, classifying the districts into red, orange and green zones, corresponding to the number of reported, ongoing cases in each district^12^. On 20th April the lock-down rules were relaxed for districts in Kerala in the green and orange zones. Subsequently, a repatriation scheme was initiated through which 127,089 Keralites had returned to the state by 30th May^13^. Passengers coming from outside Kerala were quarantined for 14 days at home or at facilities provided by the government. Local health workers monitored adherence to quarantine at the individual level.

In Phase 3, travel restrictions were implemented in red zones only; public transport restarted operations, and offices and shops were allowed to open. The active cases in Kerala increased from 16 on 8th May to 1231 on 9th June^13^, though the spread of disease through contact was restricted to ∼ 10%. The implementation of strict quarantine measures and public participation has been credited with the low rate of contact transmission.

The Kerala plan included: (1) testing the population according to World Health Organisation directives and strict quarantine of all cases suspected of infection; (2) implementation of travel bans across sub-state administrative (district) boundaries and state borders; (3) a public outreach campaign, “break the chain”, focusing on social distancing and hygiene, including mask-wearing and hand-washing; (4) use of citizen science for data collection and management^14^; (5) organisation of a special youth task force of 236,000 volunteers for supporting the vulnerable senior citizens and others under quarantine, with delivery of food, medicine and other needs^15^; and (6) arranging community kitchens to deliver meals to stranded migratory labourers and others in need. The overall approach was a decentralised and distributed one, with a clear plan of action at every level of administration, with full community engagement.

**Figure 1:**
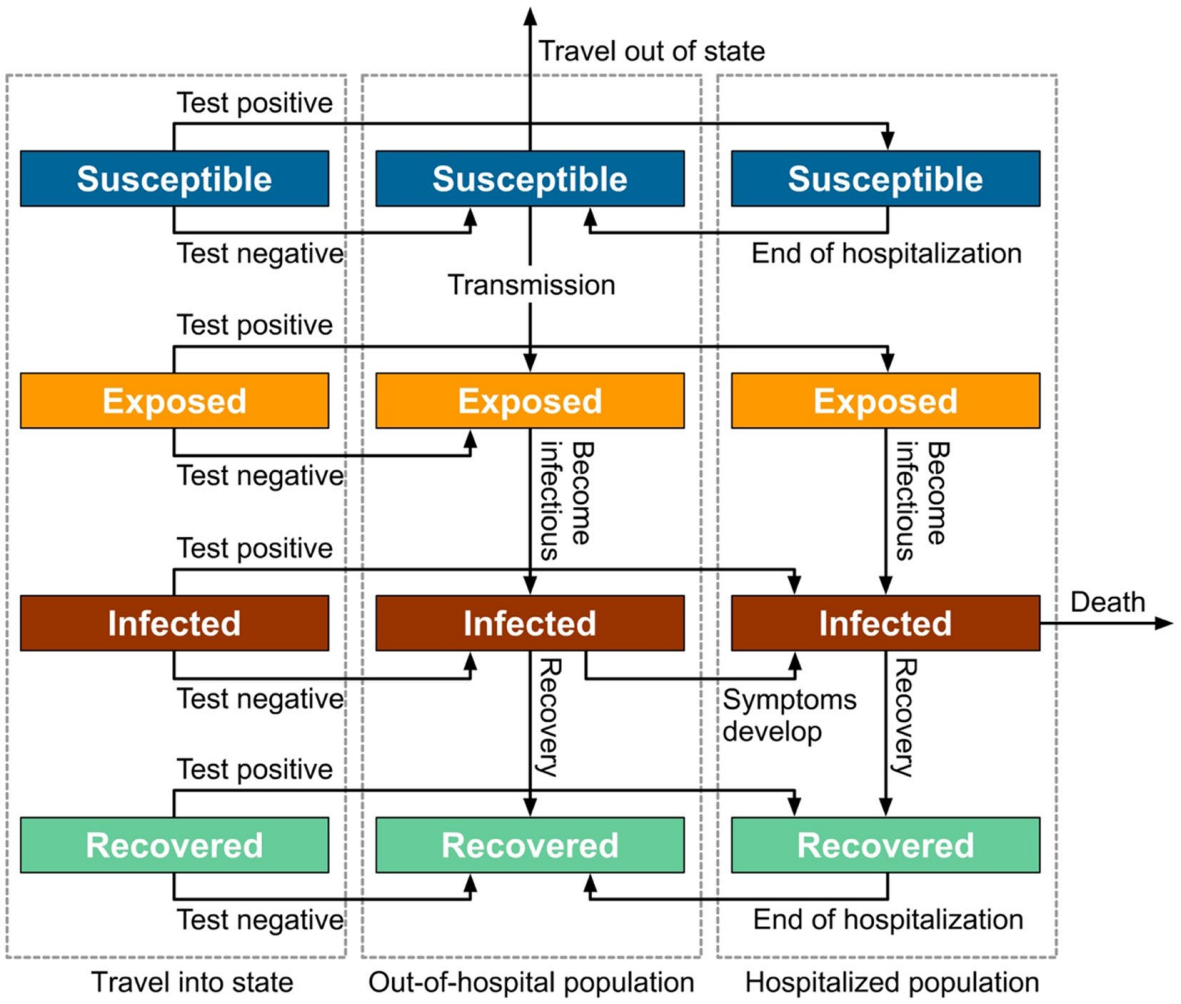
Kerala model structure. See Methods section for details.

The Kerala COVID-19 model used in this work is based on the generic class of SEIR models, with additional partitioning of the population into hospitalised and out-of-hospital compartments, with a third dealing with people travelling into the state (Fig. 1, see details in Methods section).

As models simplify the real world, certain assumptions must be made to capture the relevant behaviour effectively. The assumptions used in this work are detailed in the methods section, but we recognise that the model assumptions on the behaviour of people as an ensemble would not hold at an individual scale. Because of the novelty of the disease modelled here, aspects remain uncertain: whether asymptomatic individuals are able to transmit the virus; or whether permanent immunity is conveyed by the virus. As such information becomes available, the related assumptions in the model may be contradicted. Because of these limitations, the model is not presented as a forecast model, but rather as a tool to assess the effectiveness of certain non-pharmaceutical interventions undertaken to prevent disease transmission by giving quantitative estimates of cases and deaths due to impacts.

The data used to fit the model are cases and deaths reported via the state government. The model accounts mechanistically for some of the reporting issues with this type of data by recognising that all infected people may not be identified even when tested, but there could still be a degree of underreporting that the model might not have accounted for. Similar issues arise from the use of mortality data. However, the control of the outbreak in its early stages reduces the likelihood of underreporting in mortality during the study period.

Here we use the model to evaluate quantitatively the impact of various NPI strategies in controlling the outbreak of COVID-19 in Kerala, India. The model was fitted to the observed daily reported cases and total reported deaths to tune the model parameters. Variants of the model were then generated, to test the effects of (1) reduced testing; (2) no travel restrictions; (3) no out-of-hospital measures; (4) no in-hospital quarantine; and (5) all measures removed. Treatment of the out-of-hospital measures included consideration of the consequences of: no quarantine of out-of-hospital population with no lock-down; no track-and trace; and their combined effect (see Methods section for further details. The outputs of the model for these hypothetical cases are then analysed to assess the effectiveness of individual measures, and to examine their potential implications.

## Results

**Figure 2:**
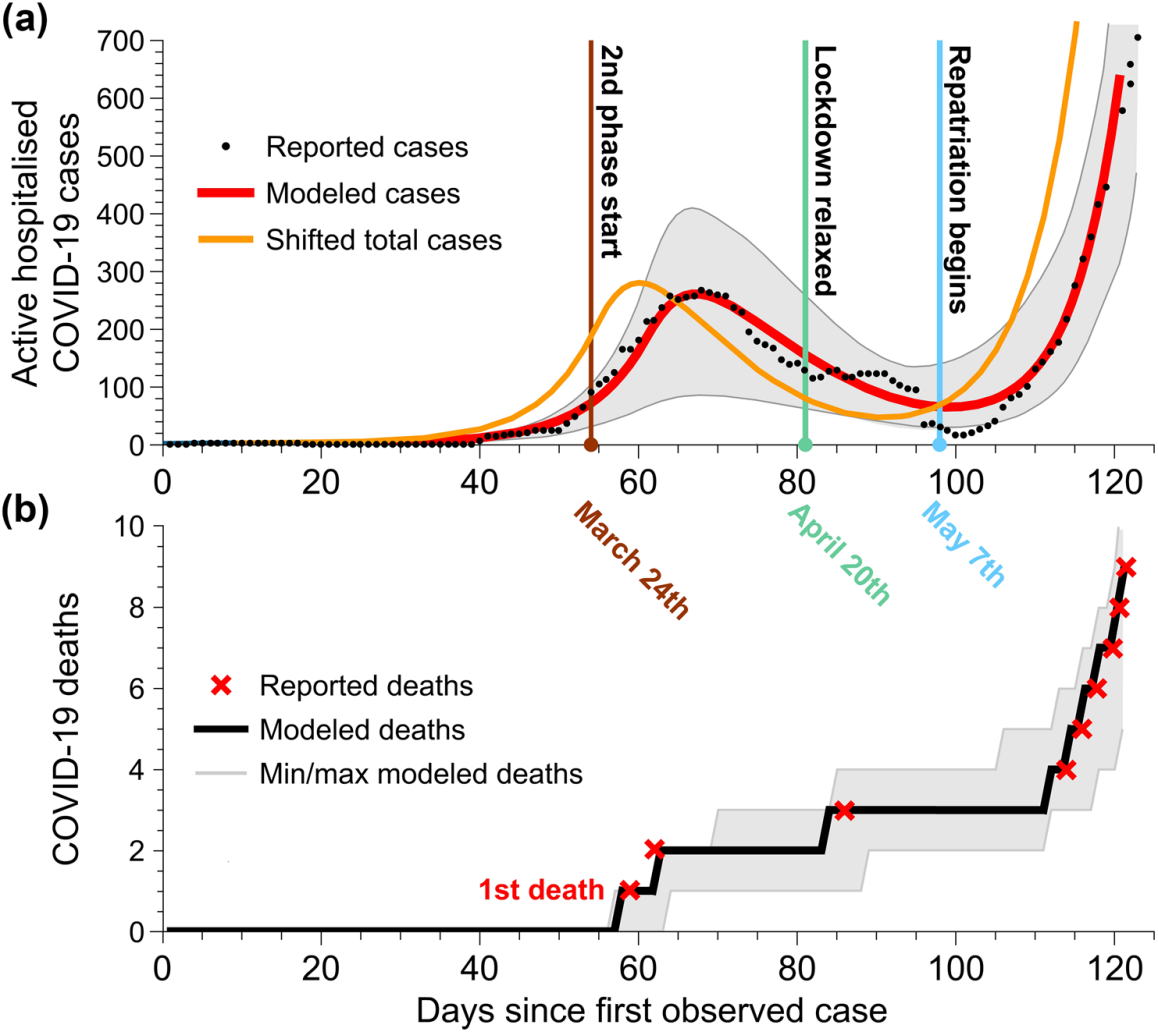
Observed and modelled COVID-19 cases in Kerala, from January 30th to May 30th 2020. (**a**) Modelled and reported active, hospitalised COVID-19 cases in the state. Also shown is the modelled total cases (in and out of hospital combined), shifted by 7 days, which is the number of days in the model between someone being suspected of having the disease and being officially reported. The minimum and maximum bounds of the modelled reported hospital cases from the MCMC fitting are included in grey. (**b**) Modelled and observed cumulative deaths due to COVID-19 in Kerala. Minimum and maximum bounds from the MCMC fitting are shown in grey.

The reference model reproduces the time series of observations of COVID-19 daily cases and deaths with high fidelity (Fig. 2). The SSR value (the sum of weighted squared residuals per observed variable^16^) for the parameter value is 858. The snapshot outputs at the end of the run, on 30th May, also correspond well with the observed hospitalised cases and reproduce the low number of deaths (see Table 1a). The number of people modelled as being in hospital at the end of the study period (30th May) are 581, with 90% CI of (447, 716), which correspond to observed cases of 624. The fitted Kerala reference model estimates a total of 8, with 90% CI of (7, 10) deaths between 30th January 2020 and 30th May 2020 (compared with 9 reported). The model admits that a proportion of infected people may not have been identified; and the 3396 with 95% CI of (3369, 3422) cumulative modelled cases (Table 1a) consist of the in-hospital infected population and the out-of-hospital, undetected, but infected population (Fig. S1). Comparison with the cumulative hospitalised cases suggests that there were many undetected cases outside the hospital as reported on 30th May. This was not always the case (Fig. 2): according to the model, there were no undetected cases in the community between day 63 (3rd April) and day 91 (1st May). Since all people who tested positive, or presented recognisable symptoms, were hospitalised, this result suggests that by the end of the simulation period, many cases were asymptomatic, or presented atypically. While it would be impossible to verify the number of out-of-hospital infected people, this result may explain why the number of cases increased rapidly, once lock-down measures were relaxed.

**Table 1:** Observations and model results.

Model	Total cases		Reported cases			
	Maximum (95% CI)	Cumulative (95% CI)	Maximum (95% CI)	Cumulative (95% CI)	Deaths (95% CI)	Mortality (%) (95% CI)
(a) *Kerala observations and model results*						
Observed	–	–	624	1208	9	0.75
Reference model	2084 (2068, 2100)	3396 (3369, 3421)	505 (501, 509)	1116 (1107, 1124)	8 (7, 8)	0.670 (0.66, 0.69)
Reduced Testing	12,617 (12,518, 12,716)	20,417 (20,252, 20,582)	2991 (2967, 3015)	6561 (6505, 6616)	45 (44, 45)	0.68 (0.67, 0.69)
No travel restrictions	21,019 (20,983, 21,056)	30,104 (30,051, 30,157)	5140 (5131, 5149)	7439 (7425, 7453)	50 (49, 50)	0.67 (0.67, 0.67)
No out-of-hospital measures	1,624,056 (1,609,944, 1,638,168)	2,530,461 (2,508,033 , 2,552,889)	590,961 (585,457, 596,464)	856,110 (848,086, 864,134)	5089 (5042, 5136)	0.59 (0.58, 0.61)
No in-hospital quarantine	17,709,110 (17,554,337, 17,863,884 )	33,300,000 (33,300,000, 33,300,000)	17,540,530 (17,529,960, 17,551,340)	31,056,110 (31,056,110, 31,056,110)	208,699 (208,690, 208,708)	0.67 (0.67, 0.67)
All measures removed	17,516,461 (17,168,606, 17,864,315)	33,300,000 (33,300,000, 33,300,000)	17,353,330 (17,318,520, 17,388,140)	31,056,220 (31,056,210, 31,056,220)	208,283 (208,265, 208,301)	0.67 (0.67, 0.67)
No track and trace	16,226,350 (16,206,950, 16,245,980)	28,408,000 (28,361,930, 28,454,070)	11,195,940 (11,121,570, 11,270,310)	14,211,250 (14,100,110, 14,322,400)	71,264 (70,874,71,653)	0.50 (0.49, 0.51)
No track and trace, no out-of-hospital measures	15,342,950 (15,320,040, 15,366,590)	32,874,741 (32,874,685, 32,874,797)	15,235,970 (15,230,870, 15,241,060)	30,645,140 (30,644,900, 30,645,380)	196,482 (196,431, 196,534)	0.64 (0.64, 0.64)
(b) *Data, for a subset of afflicted countries, for comparison*						
Country			Active	Cumulative	Deaths	Mortality (%)
Canada (35.2 M) 26/01/2020			35,992	91,667	7158	7.8
Egypt (100 M) 14/02/2020			16,843	23,449	913	3.9
Germany (83 M) 27/01/2020			9751	183,189	8530	4.7
Italy (60 M) 31/01/2020			43,691	232,664	33,340	14.3
India (1380 M) 30/01/2020			89,706	181,827	5185	2.9
New Zealand (4.8 M) 28/02/2020			1	1504	22	1.5

(a) Observations and results from the reference model run, along with runs varying the level of state intervention. Mortality is calculated as the ratio of deaths to cumulative hospitalised cases. The maximum corresponds to the number of people modelled as being in hospital at the end of the study period. (b) Observations from other countries, and India including Kerala, for comparison. All snapshots are for 30th May, 2020, with the date of introduction given in the first column. The total population of each country is given in parentheses in the first column, in units of millions (M). Timeseries for these countries are shown in Fig. S3.

As of June 2020, the state had 1280 public hospitals and 2062 private hospitals, giving a state-wide total of 99,227 hospital beds, of which 4961 were intensive care unit beds, with 2481 ventilators^17^. As of 30th May 2020, Kerala had not reached any of these thresholds. However, it shows a trend of the number of cases increasing from day 92, indicating both an increase in transmission rate as well as the intake of already-infected people into the state. Though the effective reproduction number ${\mathcal{R}}_{e}$ had dropped to 0.10 by 24th March, a value well below the threshold of one required for the number of cases to decline, it had risen far above the threshold value by 20th April, reaching 2.2 (greater than its initial value 1.9 at the beginning of simulation, see Supplementary Material, Table S3). This is consistent with reports in mid-July, according to which community transmission was responsible for more newly-reported cases than the influx of infected people.

**Figure 3:**
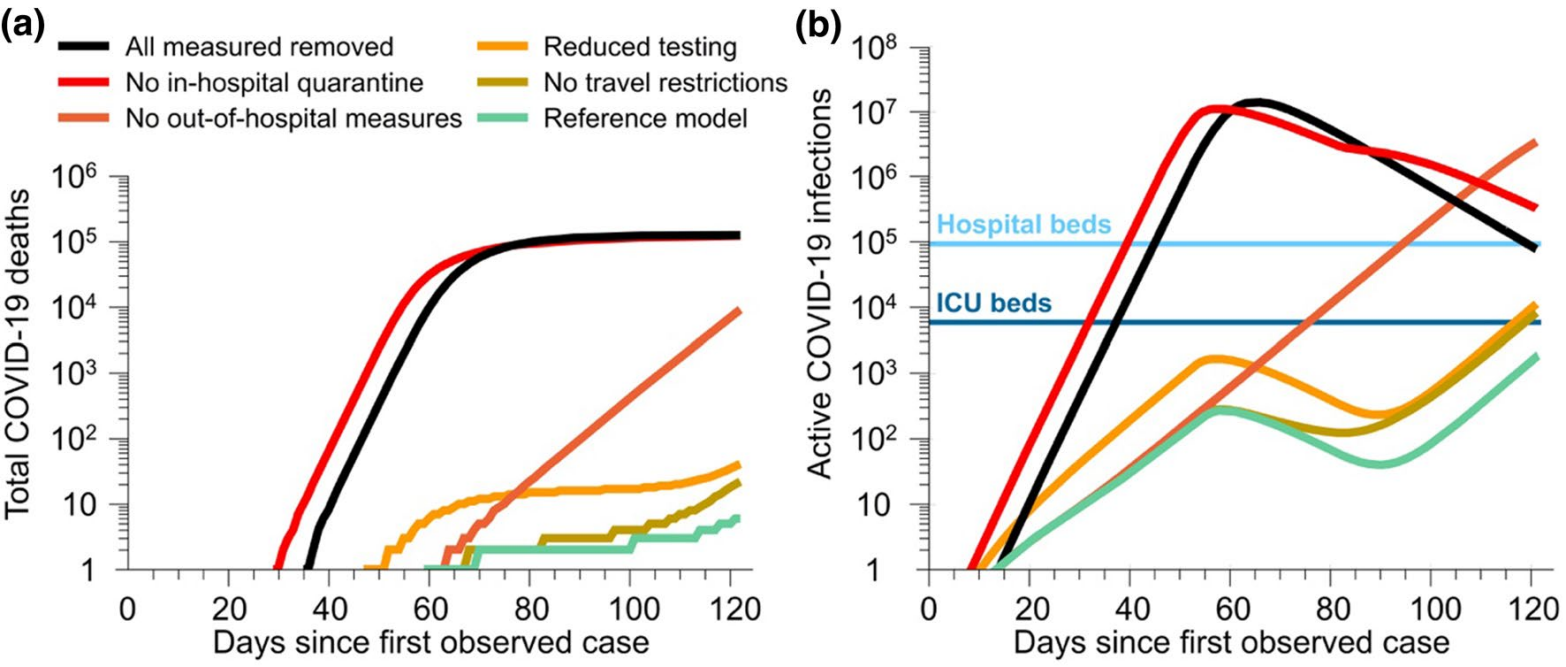
Results from the exploration of government measures. (**a**) Compares the deaths due to COVID-19 in the state, and (**b**) compares the active COVID- 19 cases in Kerala. The number of hospital beds and ICU beds available in Kerala are also shown.

In the simulations which considered the impact of government measures, removing in-hospital quarantine had the most effect, with only the removal of all modelled government actions yielding a higher total number of cases and deaths (see Table 1a and Fig. 3 and Fig. S2). Removing out-of-hospital measures also had a high impact on both death and cases. Increasing ${\mathcal{R}}_{e}$ (to simulate no track and trace) augmented the rate of increase, bringing it closer to the case of no in-hospital quarantine. Reduced testing and increasing the influx of people into the state also augmented infections and deaths, but at a less alarming rate than the no-quarantine cases.

Hence, the considerable effort by volunteers, health workers, government departments and the general public to enact the full quarantine appear to have been effective, according to the model. Quarantining had reduced the transmission rate and stopped the spread from within hospital to out-of-hospital, as well as within the out-of-hospital population. Relaxing or removing quarantine would have overstretched Kerala’s hospital facilities.

According to the reference model, only a small proportion of the population had had the virus (2281 with 90% CI of (1306, 3540)) by 30th May. This was largely due to the success of the initial rapid actions taken by the government, with strong support from the local community to contain the spread of the disease. These low numbers do not come close to the rate required for herd immunity (60% for an ${\mathcal{R}}_{0}$ of 2.5^18^, or 47% for an ${\mathcal{R}}_{0}$ of 1.9 which occurred with track-and-trace) so that population immunity cannot be relied upon to slow infection.

Without further decrease in the transmission rate, the outbreak, which was successfully stalled, has the potential to return in full force. For comparison with other countries, a snapshot at the end of May (Table 1b) shows a high level of success in Kerala, in keeping the number of infections down. However, it is evident from the inferred ${\mathcal{R}}_{e}$ that relaxation came too early, and that longer-term control strategies are needed to prevent further escalation through community transmission^19^.

## Discussion

The leadership response at various levels of government when COVID-19 was first detected in Kerala was well-coordinated and prompt, and benefitted from a strong reputation built on a track record of successfully dealing with previous health emergencies^20^. As a consequence, there was a high level of cooperation from all relevant government departments as well as the well-informed population, and a high degree of adherence to the government measures. This was evident from the newspaper reports of the time^21–23^, which showed that reported cases of non-adherence were low. This made it straightforward to model the impact of various government measures on the propagation of the disease: in the event of poor adherence, the effectiveness of the measures would have been difficult to discern using a model with transmission rates changing in line with these measures. Once the model parameters were tuned to fit the data, the reference model could be run for hypothetical cases in which the various government measures had not been enacted.

The data and the model show remarkably low cases and fatalities (a total of three) until 7th May, when the lock-down was eased in districts with low case numbers and repatriation of Keralites stranded outside the state began, which was followed by a period of exponentially growing infection till the end of the study period, by which time the number of fatalities had increased to 9. Compared with many other countries which had similar dates of introduction of the disease, the fatalities are very low.

By using the model to simulate what the consequences would have been if the government had not acted promptly, we see that in the worst case of no government action the entire population would have become infected at some point, and the total fatality would have risen to over two hundred thousand within the four-month study period. In this worst-case scenario, and many of the hypothetical scenarios, Kerala’s medical facilities and volunteer activities would have been over-stretched, rapidly increasing the death toll beyond what is modelled here. Note that stringent track-and-trace measures were in place from the very beginning of the outbreak in Kerala, making it difficult to evaluate the impact of this particular measure. We can only speculate that the ${\mathcal{R}}_{e}$ value at the beginning of the study period would have been significantly higher otherwise, see for example values greater than 3 reported from other regions^24,25^.

During the period of study, the number of deaths relative to the total number of reported infected people was 0.7%. It had since decreased to 0.4% as of 10th July^26^, though the number of cases had increased to 6,951. The case fatality rate is low (*cf*. 2.7% for India as a whole, 15.5% for the UK, and 4.3% for the USA, according to the Johns Hopkins Coronavirus Resource Centre^7^ on 10th July 2020). The role played by Kerala’s public healthcare system (which in itself is indicative of the foresight of multiple governments in building Kerala’s healthcare system over the last few decades) cannot be overlooked when analysing the low death rate in Kerala. This consideration emphasises the importance of a combination of long-term planning, as well as short-term, rapid response. Other factors (such as population demographics) could have contributed to the low mortality, but they fall outside the scope of this work.

At the end of Phase-2, lock-down rules were further relaxed, leading to increased rates of infection. Hence, the measures have not succeeded in solving the problem, only delayed it. However, that in itself is significant for a densely-populated state such as Kerala, within a developing country. By successfully keeping the number of infected cases low in the initial four months, Kerala ensured that its medical facilities were not stretched beyond breaking point. At the end of the study period, Kerala is starting from a smaller number of infected people than many countries of comparable size, or indeed many of the other Indian states (see Table 1b and Supplementary Materials Fig. S3), even though Kerala was the first state in India to report a case of COVID-19.

This achievement has allowed the state to (a) scale-up COVID-19 isolation and treatment facilities; (b) mobilise a massive volunteer force to help people adversely affected by the disease and government measures; (c) put in place aid delivery mechanisms for workers who had lost their jobs and their income; and (d) inform the population of the dangers of the pandemic and of the importance of modifying social behaviour patterns to avoid community transmission. What remains unknown at present is whether the psychological pressures, brought on by the sustained threat on health, many months of social distancing, and financial hardship, could in turn lead to break down in discipline.

As government measures relaxed, the burden shifted more on to the public to maintain the principles embedded in Kerala’s “break the chain” campaign^27,28^, designed to increase awareness of the importance of handwashing, mask wearing and social distancing for slowing the transmission of the virus, for as long as the coronavirus threat remained. If the population is unable to break the chain, then the alternatives are limited: it would be either return to lock down, or head towards a major crisis, according to the model simulation.

The Kerala model highlights the importance of strong leadership in a crisis, working together with a dedicated and committed body of healthcare workers and a literate and cognisant society; the value of full community engagement to fight the danger; the importance of a public healthcare system that is affordable, agile and flexible; and the need for long-term commitment to building health care facilities.

The initial part of the study period demonstrates the effectiveness of the actions adopted by a developing society, with low cases and deaths. In the wake of the pandemic, many analyses will be undertaken to determine whether various governments took the right path to dealing with COVID-19. Several strategies will be examined. The Kerala COVID-19 response is worthy of consideration in the comparisons, not only because it flattened the curve in the early days against all odds, but also because of the secondary period of exponential growth of cases in the subsequent, post-lock-down months.

## Methods

The Kerala COVID-19 model presented here is based on the generic class of susceptible-exposed-infected-recovered (SEIR) models, with additional partitioning of the population into compartments dealing with hospitalised (*h*); out-of-hospital (*o*) and travel into state (*δ*). Symbols and definitions used here for all model variables are listed in Table S1 and for all model parameters are provided in Table S2. We first examine the out-of-hospital compartment.

### Out-of-hospital compartment

In the model, the rate of change in the out-of-hospital, susceptible population (*S*_*o*_) is expressed as:

1 $$\frac{{{\text{d}}S_{o} }}{{{\text{d}}t}} = - \frac{{\lambda S_{o} I_{o} }}{{H_{o} }} + \omega S_{h} + \mu_{sp} \delta_{S} - \delta_{T} ,$$

where *S*_*o*_, the out-of-hospital, susceptible compartment, decreases as some members of the pool move to the out-of-hospital, exposed compartment *E*_*o*_, at the rate of (*λI*_*o*_*/H*_*o*_), according to their interaction with infected, out-of-hospital people (*I*_*o*_) within a total population (*H*_*o*_) in the out-of-hospital pool, and the rate of transmission *λ*. The population *S*_*o*_ increases due to a fraction of hospitalised susceptible population (*S*_*h*_) leaving hospital, with the rate of transfer determined by *ω*, which is the reciprocal of the period at the end of which a person is released from hospital, if free of symptoms. The susceptible people travelling into Kerala, *δ*_*S*_, who tested negative for COVID-19 also move into the *S*_*o*_ pool, with the rate of transfer determined by *μ*_*sp*_, the COVID-19 test specificity. The total population is held constant through travel out of the state^29^, which is assumed to be equivalent to the total population *δ*_*T*_ entering the state, where *δ*_*T*_ = *δ*_*S*_ + *δ*_*E*_ + *δ*_*I*_ + *δ*_*R*_.

The dynamics of the exposed population in the out-of-hospital compartment, *E*_*o*_, are given by:

2 $$\frac{{{\text{d}}E_{o} }}{{{\text{d}}t}} = \frac{{\lambda S_{o} I_{o} }}{{H_{o} }} - pE_{o} + \left( {1 - \mu_{se} } \right)\delta_{E} ,$$

in which a part of the out-of-hospital susceptible population *S*_*o*_ is transferred to *E*_*o*_ when exposed to the disease, but prior to developing any symptoms, as represented by the term *λS*_*o*_*I*_*o*_*/H*_*o*_. People leave the compartment when they become infectious, moving to the out-of-hospital infected population, with this rate of transfer determined by *p*, the rate at which exposed people become infectious. Exposed travellers into the state who tested negative for COVID-19 (false negative) also add to the E_o_ pool through the term (1 − *μ*_*se*_)*δ*_*E*_, where *μ*_*se*_ is the COVID-19 test sensitivity.

The rate of change in the infected, but out-of-hospital pool *I*_*o*_ is computed as:

3 $$\frac{{{\text{d}}I_{o} }}{{{\text{d}}t}} = pE_{o} + \left( {1 - \mu_{se} } \right)\delta_{I} - rI_{o} - \sigma I_{o} ,$$

where *I*_*o*_ increases when people from the out-of-hospital exposed compartment become infectious, at a rate *p*. The pool size also increases when travellers who do not test positive for COVID-19 enter the state ((1 − *μ*_*se*_)*δ*_*I*_). This pool decreases when people recover from the disease at a rate *r*, the recovery rate, progressing to the out-of-hospital recovered population. The out-of-hospital infected population also decrease when members move to the hospitalised infected population when they develop symptoms identifiable as COVID-19, at a rate *σ*, the rate at which infected people develop noticeable symptoms. As the symptomatic cases are removed from the population, along with the asymptomatic cases which tested positive, the transmission rate approximates transmission from asymptomatic, untested cases only.

The rate of change in the fourth pool *R*_*o*_, in the out-of-hospital compartment representing the recovered population, is estimated as:

$$\frac{{{\text{d}}R_{o} }}{{{\text{d}}t}} = rI_{o} + \omega R_{h} + \mu_{sp} {\updelta }_{R} \, .$$

The compartment *R*_*o*_ represents people who have had COVID-19 and recovered, and were afterwards released from hospital. This population grows when out-of- hospital infectious people recover (*rI*_*o*_); when the hospitalised recovered people (*R*_*h*_) are released from hospital at the rate *ω*, the reciprocal of the period from first negative test to release from hospital; and when recovered people travel into the state and test negative for the virus (*μ*_*sp*_*δ*_*R*_).

### Hospitalised compartment

The compartment *S*_*h*_, representing hospitalised people who have not been exposed to the virus, is modelled as:

5 $$\frac{{{\text{d}}S_{h} }}{{{\text{d}}t}} = \left( {1 - \mu_{sp} } \right){\updelta }_{S} - \omega S_{h} ,$$

in which the pool increases when travellers come into the state, and receive false-positive for COVID-19 test results ((1 − *μ*_*sp*_)*δ*_*S*_), and decreases when people test negative for COVID-19 and are released after a period of (1/*ω*) days. It is assumed that hospitalised individuals are unable to contract the virus, implying a fully effective quarantine.

The hospitalised exposed population (*E*_*h*_) dynamics are modelled as:

6 $$\frac{{{\text{d}}E_{h} }}{{{\text{d}}t}} = \mu_{{{\text{se}}}} {\updelta }_{E} - pE_{h} ,$$

where increases in the compartment result from travellers into the state correctly testing positive for COVID-19 (*μ*_*se*_*δ*_*E*_) and decreases result from people becoming infectious and progressing to the hospitalised, infected, population (*I*_*h*_), at rate *p*.

The change in the hospitalised, infected, population (*I*_*h*_) is modelled as:

7 $$\frac{{{\text{d}}I_{h} }}{{{\text{d}}t}} = pE_{h} + \sigma I_{o} + \mu_{se} {\updelta }_{I} - rI_{h} - D,$$

where this pool increases when the hospitalised exposed population becomes infectious (*pE*_*h*_); when people in the out-of-hospital infected pool develop symptoms and are hospitalised (*σI*_*o*_); and from travellers into the state correctly testing positive (*μ*_*se*_*δ*_*I*_). Population in this pool decrease with recoveries (*rI*_*h*_), and from deaths (*D*). Deaths are modelled as occurring only in the hospitalised population, as the symptoms of COVID-19 are expected to be severe enough to be detectable prior to patient mortality.

Finally, the hospitalised recovered population (*R*_*h*_) is modelled as:

8 $$\frac{{{\text{d}}R_{h} }}{{{\text{d}}t}} = rI_{h} - \omega R_{h} + \left( {1 - \mu_{sp} } \right)\delta_{R} ,$$

where increases result from recovery of hospitalised infected people (*rI*_*h*_) and from entry of recovered people into the state ((1 − *μ*_*sp*_)*δ*_*R*_). Decreases were from people leaving hospital, having tested negative for 1/ω days.

### Implementation

The total number of people travelling into the state per day, *δ*_*T*_, was modelled as an independently distributed normal random variable with mean 46,000 and standard deviation 2000^30^, until 24th March 2020, when travel restrictions into the state began. After this date the number of people entering the state was reduced to 48,715 total people during the entire period from 24th March to 14th May 2020^31^, mainly consisting of non-resident Keralites returning to the state in repatriation efforts. The total number of people entering the state is modelled as a uniformly distributed random variable in the interval [0, 1840] for this period. From 16th May 2020, estimates for the number of people travelling into the state are available^13^, and so are used as inputs to the model.

The number of non-susceptible people travelling to the state, *δ*_*T*_* − δ*_*S*_, is modelled as a binomially-distributed random variable with number of trials equal to the number of people travelling into the state, and probability of success equal to the date-dependent global COVID-19 incidence rate^1^. The numbers of exposed, infected and recovered people travelling into the state (*δ*_*E*_, *δ*_*I*_ and *δ*_*R*_) are then uniformly distributed such that *δ*_*T*_* − δ*_*S*_ = *δ*_*E*_ + *δ*_*I*_ + *δ*_*R*_, (${\updelta }_{E} \sim uniform\left( {0,{\updelta }_{T} - {\updelta }_{s} } \right)$, ${\updelta }_{{\text{I}}} \sim uniform\left( {0,{\updelta }_{T} - {\updelta }_{S} - {\updelta }_{E} } \right)$, ${\updelta }_{R} = {\updelta }_{T} - {\updelta }_{S} - {\updelta }_{E} - {\updelta }_{I} )$ . The number of deaths were also calculated stochastically, as a binomially-distributed random variable with *I*_*h*_ trials, and probability *d,* $D\sim binomial\left( {I_{h} ,d} \right)$.

The actions taken by the state of Kerala led to changes in transmission rates, according to the model. Hence the transmission rate is modelled by the piece-wise function

9 $$\lambda \left( t \right) = \left\{ {\begin{array}{*{20}l} {\lambda_{1} , \;30\;\mathrm{January}\;2020 < t \le 24\;\mathrm{March}\;2020 } \hfill \\ {\lambda_{2} ,\; 24\;\mathrm{March}\;2020 < t \le 20\;\mathrm{April}\;2020} \hfill \\ {\lambda_{3} , \;20\;\mathrm{April}\;2020 < t \le 30\;\mathrm{May}\;2020.} \hfill \\ \end{array} } \right.$$

Note that Phase 2 is split into two stages on 20th April, because of the relaxations in lock-down on that day. A delay in reporting of ongoing hospitalised cases (*t*_*d*_) is also set at 7 days, to reflect delays in updating of statistics due tohe time required for testing, and other uncertainties. Similar delays have been found in other COVID-19 models^32^, as the novelty of the disease means testing delays affect every afflicted area.

The model is run from 30th January 2020 to 30th May 2020, assuming an initial population of 33.3 million susceptible, out-of-hospital people. Since there are stochastic components to the model (the number of people entering the state; the number of infected people; and the number of deaths per day), the model is run 300 times and the mean values for each compartment at each time point taken, such that the results presented constitute an ensemble mean. The sensitivity of the results to the number of times the model is run is shown in the supplementary material Fig S4.

### Model assumptions

The susceptible-infected-recovered (SIR) modelling framework, of which the model above is a variation, has inherent assumptions^33–35^. Furthermore, there are other assumptions introduced here.

It is assumed here that the identification of people entering the state is complete, and that testing is carried out on all people entering the state. This is unlikely to be true: there are always limits to testing capacity, such that numbers entering the state above the limit cannot be tested. This could have occurred prior to the travel ban implemented on 24th March 2020. People entering the state may also go unidentified when checkpoints are avoided, or provide incomplete information on travel history.

Similarly, the model assumes that quarantining of hospitalised people is highly successful, so no one in hospital interacts with those not hospitalised. In this ideal view of quarantine, there is no spread from those in hospital to those outside. However, this may not always hold, as those in hospital may come into contact with out-of-hospital people, for example through healthcare workers in hospitals. Efforts have been taken in Kerala to reduce the spread in such environments, by designating entire government-run hospitals as COVID-only hospitals, and by providing essential personal protective equipment to all staff within those hospitals.

Quarantining of people entering the state, and those suspected of coming into contact with infected people was an important component of the containment strategy implemented in Kerala. Within the model this is implemented implicitly, as changes in transmission rate on 24th March 2020 and 20th April 2020.

Tracking and tracing of people who came into contact with potentially infectious people was also a key part of the Kerala plan. This was implemented from the very beginning of the virus’s introduction into Kerala. In the model there is no explicit description of this, but appears as a reduction in the transmission rate *λ* from the outset, and changes to the value of *σ*, which depends on the identification of individuals with severe symptoms.

As the state enacted the track and trace system from the identified initial introduction of COVID-19, it is not possible to judge the impact the scheme has had on containment. There are no data on the disease dynamics without track and trace for the state, so no estimate on the changes to *σ* and *λ* can be estimated, and the impact of its removal is not possible to quantify with this model.

### Reproduction number

The potential for a contagion to spread is often expressed as ${\mathcal{R}}_{0}$, the basic reproduction number, which represents the expected number of cases that might be infected by a single case, given all the members of the population are susceptible^24^, and no deliberate interventions against disease transmission have been taken^36^. The ${\mathcal{R}}_{0}$ number of the model can be computed given three of the SEIR parameters—${\uplambda }_{0}$, *r* and *σ*—as:

10 $${\mathcal{R}}_{0} = \frac{{{\uplambda }_{0} }}{{r + {\upsigma }}},$$

where ${\uplambda }_{0}$ represents the unknowable transmission rate in a population with no interventions. The effective reproduction number, ${\mathcal{R}}_{e}$, represents the mean number of new infections caused by an infected individual at a given time. In the presented model:

11 $${\mathcal{R}}_{e} = \frac{{\lambda_{{\text{i}}} }}{{r + {\upsigma }}}\,\frac{S}{H},$$

for $i = 1,2,3$, the basic reproduction number scaled by the proportion of the population who are still susceptible. As the total non-susceptible population remains low within the study the effective reproductive number can be approximated by:

12 $${\mathcal{R}}_{e} = \frac{{\lambda_{{\text{i}}} }}{{r + {\upsigma }}},$$

for $$i = 1,2,3$$.

### Fitting model parameters

The Kerala COVID-19 model was fit using the FME package in the R programming language^37^, which implements a Bayesian Monte-Carlo Markov Chain (MCMC) method.

The model was fit to the daily, hospitalised, ongoing cases, and total deaths, reported by the state government^13^.

A constrained MCMC simulation was implemented, using the adaptive Metropolis algorithm, with normally distributed likelihood^16^ (for full details see the supplementary information). The method is implemented in the FME package, weighted to the reciprocal of the standard deviation of the observed data, to fit the model parameters {*λ*_1_, *λ*_2_, *λ*_3_, *σ*, *d*}. Parameters were constrained to ${\mathbb{R}}^{+}$, with parameters representing probabilities constrained to the interval [0,1]. Initial runs were carried out to establish maximal step-lengths to keep acceptance rates approximately 23.4%^38^.

An MCMC chain of 600,000 steps was run to provide parameter estimates, which gave SSR values in the range [858, 892]. Convergence was judged graphically (see Fig. S5) and using the multivariate effective sample size^39^. The minimum effective sample size of the 5-parameter problem is 8605 at 5% tolerance level^40,41^. The 600,000-step chain achieved an effective sample size of 19,047, hence the chain was judged to contain sufficient information for stable estimation at the 5% tolerance level. The final parameters taken were those which gave the lowest SSR, as detailed in Table S3 with upper and lower bounds. Full parameter distributions are shown in Fig. S6.

The number of active cases is published daily, accounting for cases undergoing treatment or monitoring in the government hospitals^13^. This includes all those who have tested positive, or displayed moderate to severe symptoms of COVID-19, and may include those who do not have the virus. Hence, the full hospitalised population (*S*_*h*_ + *E*_*h*_ + *I*_*h*_ + *R*_*h*_) is fit to the observed number of active cases. Total mortality caused by COVID-19 is also published in daily bulletins by the government of Kerala^13^. All deaths recorded as COVID-19 have tested positive for COVID-19.

The fitted parameter values are shown in Table S3, along with the inferred ${\mathcal{R}}_{e}$ values. The fitted model is treated as the reference model.

### Hypothetical cases exploring effectiveness of government measures

The fitted version of the model is treated as a reference. The model was then modified to explore how the various measures implemented by the Kerala government impacted the spread of COVID-19 in Kerala. Variants of the model, in which the various government measures were removed, were run from 30th January 2020 to 30th May 2020, and the number of modelled deaths due to COVID-19 in each variant case was compared against the reference model results, yielding the number of extra deaths that would have resulted, had the government not enacted the measures for reducing the spread of COVID-19.

The reference Kerala model described above combines hospitalisation and quarantine, with testing and tracing, restrictions on travel and lock-down within the state. To quantify the impact of each of these measures, they were removed individually, and in combination, and the impacts evaluated from the variant model runs.

### Reduced testing

To model reduced testing of people entering the state, a testing parameter $a$ was introduced such that the equations become:

13 $$\frac{{{\text{d}}S_{o} }}{{{\text{d}}t}} = - \frac{{\lambda S_{o} I_{o} }}{{H_{o} }} + \omega S_{h} + \left( {a\mu_{sp} + \left( {1 - a} \right)} \right)\delta_{S} - \delta_{T} ,$$

14 $$\frac{{{\text{d}}E_{o} }}{{{\text{d}}t}} = \frac{{\lambda S_{o} I_{o} }}{{H_{o} }} - pE_{o} + \left( {a\left( {1 - \mu_{se} } \right) + \left( {1 - a} \right)} \right)\delta_{E} ,$$

15 $$\frac{{{\text{d}}I_{o} }}{{{\text{d}}t}} = pE_{o} + \left( {a\left( {1 - \mu_{se} } \right) + \left( {1 - a} \right)} \right)\delta_{I} - rI_{o} - \sigma I_{o} ,$$

16 $$\frac{{{\text{d}}R_{o} }}{{{\text{d}}t}} = rI_{o} + \omega R_{h} + \left( {a{\upmu }_{sp} + \left( {1 - a} \right)} \right)\delta_{R} ,$$

17 $$\frac{{{\text{d}}S_{h} }}{{{\text{d}}t}} = a\left( {1 - \mu_{sp} } \right)\delta_{S} - \omega S_{h} ,$$

18 $$\frac{{{\text{d}}E_{h} }}{{{\text{d}}t}} = a\mu_{se} \delta_{E} - pE_{h} ,$$

19 $$\frac{{{\text{d}}I_{h} }}{{{\text{d}}t}} = pE_{h} + \sigma I_{o} + a\mu_{se} {\updelta }_{I} - rI_{h} - D,$$

and

20 $$\frac{{{\text{d}}R_{h} }}{{{\text{d}}t}} = rI_{h} - \omega R_{h} + a\left( {1 - \mu_{sp} } \right)\delta_{R} .$$

The testing rate *a* was 100% in the reference run and was then reduced to 10% in the hypothetical case considered here, to represent a highly inefficient testing system. Further examples of the impact of the testing rate value are shown in Fig. S7.

This presumes that in the reference run, the system in place is 100% effective, and that all people entering the state are tested. Note that a lower testing rate in the reference run would also change the fit, resulting in a higher value of *λ* and consequently a higher ${\mathcal{R}}_{\mathrm{e}}$ number.

### No travel restrictions

To represent the system with no restrictions of travel into the state, *δ*_*T*_ is set to pre-outbreak levels, and for the entire modelling period *δ*_*T*_ is treated as a normal random variable with mean 46,000 and standard deviation 2,000, with the number of non-susceptible people entering determined by the time-dependent global COVID-19 incidence^1^.

Keeping *δ*_*T*_ at pre-outbreak levels implies there is no reduction in travel to Kerala during the outbreak. While this might have been possible, travel bans to afflicted areas had been implemented by some countries, which could have reduced visitor numbers to Kerala, even in the absence of any controls on this imposed by the Kerala government. Such a reduction is not dealt within this model run.

### No out-of-hospital measures

The quarantining of out-of-hospital population is treated implicitly in the reference model, with values of the transmission rate *λ* decreasing by 24th March, the beginning of the lock-down phase. In the variant run in which we assume there was no quarantine outside of hospital, the transmission rate *λ* was held constant at the pre-lock-down value *λ*_*1*_.

The results of this run therefore represent the impact of no lock-down with no out-of-hospital quarantining. This presumes there was no change in behaviour of the population in response to the state interventions, and that the ${\mathcal{R}}_{e}$ number remained at 1.9 throughout the modelled period.

Another aspect of no out-of-hospital control is that no track-and-trace measures would have been implemented. It is difficult to judge what the effect of removing this measure might have been, since track-and-trace measures were implemented in Kerala from the very first day, and could have contributed to the relatively-low ${\mathcal{R}}_{e}$ value of 1.9 inferred here, compared with values between 3 and 5.7 reported for early days of COVID-19^21^. Therefore, we also carried out a simulation in which the initial ${\mathcal{R}}_{e}$ value was raised somewhat arbitrarily to 3, and the subsequent ${\mathcal{R}}_{e}$ values were increased by the same proportion (see Table S3). We also ran a simulation in which ${\mathcal{R}}_{e}$ of 3 was maintained throughout the simulation period, as exemplifying the case in which there was no track-and- trace, no lock-down and no out-of-hospital quarantine.

### No in-hospital quarantine

To model the outcome of a COVID-19 outbreak wherein the quarantining of hospitalised individuals was ineffective, the equations were changed to allow mixing between the in-hospital and out-of-hospital populations. Hence the model equations become:

21 $$\frac{{{\text{d}}S_{o} }}{{{\text{d}}t}} = - \frac{{\lambda S_{o} \left( {I_{o} + I_{h} } \right)}}{{H_{o} + H_{h} }} + \omega S_{h} + \mu_{sp} \delta_{S} - \delta_{T} ,$$

22 $$\frac{{{\text{d}}E_{o} }}{{{\text{d}}t}} = \frac{{\lambda S_{o} \left( {I_{o} + I_{h} } \right)}}{{H_{o} + H_{h} }} - pE_{o} + \left( {1 - \mu_{se} } \right)\delta_{E} ,$$

23 $$\frac{{{\text{d}}I_{o} }}{{{\text{d}}t}} = pE_{o} + \left( {1 - {\upmu }_{se} } \right)\delta_{I} - rI_{o} - \sigma I_{o} ,$$

24 $$\frac{{{\text{d}}R_{o} }}{{{\text{d}}t}} = rI_{o} + \omega R_{h} + \mu_{sp} \delta_{R} ,$$

25 $$\frac{{{\text{d}}S_{h} }}{{{\text{d}}t}} = \left( {1 - \mu_{sp} } \right)\delta_{S} - \omega S_{h} - \frac{{\lambda S_{h} \left( {I_{o} + I_{h} } \right)}}{{H_{o} + H_{h} }},$$

26 $$\frac{{{\text{d}}E_{h} }}{{{\text{d}}t}} = {\upmu }_{se} \delta_{E} - pE_{h} + \frac{{\lambda S_{h} \left( {I_{o} + I_{h} } \right)}}{{H_{o} + H_{h} }},$$

27 $$\frac{{{\text{d}}I_{h} }}{{{\text{d}}t}} = pE_{h} + \sigma I_{o} + \mu_{se} \delta_{I} - rI_{h} - D,$$

and

28 $$\frac{{{\text{d}}R_{h} }}{{{\text{d}}t}} = rI_{h} - \omega R_{h} + \left( {1 - \mu_{sp} } \right)\delta_{R} .$$

In this set of simulations, we explore theoretically the effect of complete break-down in the quarantine of hospitalised population. This hypothetical case could occur if the hospital staff were not wearing appropriate personal protective equipment, or insufficient safety procedures were put in place for workers and non-COVID-19 patients.

### All measures removed

The final variation considers an outbreak where no action was taken by the state of Kerala to prevent the spread of the disease. This is modelled by combining the variant implementations above, such that the model equations become

29 $$\frac{{{\text{d}}S_{o} }}{{{\text{d}}t}} = - \frac{{\lambda S_{o} \left( {I_{o} + I_{h} } \right)}}{{H_{o} + H_{h} }} + \omega S_{h} + \left( {a\mu_{sp} + \left( {1 - a} \right)} \right)\delta_{S} - \delta_{T} ,$$

30 $$\frac{{{\text{d}}E_{o} }}{{{\text{d}}t}} = \frac{{\lambda S_{o} \left( {I_{o} + I_{h} } \right)}}{{H_{o} + H_{h} }} - pE_{o} + \left( {a\left( {1 - \mu_{se} } \right) + \left( {1 - a} \right)} \right)\delta_{E} ,$$

31 $$\frac{{{\text{d}}I_{o} }}{{{\text{d}}t}} = pE_{o} + \left( {a\left( {1 - \mu_{se} } \right) + \left( {1 - a} \right)} \right)\delta_{I} - rI_{o} - \sigma I_{o} ,$$

32 $$\frac{{{\text{d}}R_{o} }}{{{\text{d}}t}} = rI_{o} + \omega R_{h} + \left( {a\mu_{sp} + \left( {1 - a} \right)} \right)\delta_{R} ,$$

33 $$\frac{{{\text{d}}S_{h} }}{{{\text{d}}t}} = a\left( {1 - \mu_{sp} } \right)\delta_{S} - \omega S_{h} - \frac{{\lambda S_{h} \left( {I_{o} + I_{h} } \right)}}{{H_{o} + H_{h} }},$$

34 $$\frac{{{\text{d}}E_{h} }}{{{\text{d}}t}} = a\mu_{se} \delta_{E} - pE_{h} + \frac{{\lambda S_{h} \left( {I_{o} + I_{h} } \right)}}{{H_{o} + H_{h} }},$$

35 $$\frac{{{\text{d}}I_{h} }}{{{\text{d}}t}} = pE_{h} + \sigma I_{o} + a\mu_{se} \delta_{I} - rI_{h} - D,$$

and

36 $$\frac{{{\text{d}}R_{h} }}{{{\text{d}}t}} = rI_{h} - \omega R_{h} + a\left( {1 - \mu_{sp} } \right){\updelta }_{R} .$$

The transmission rate *λ* is kept constant at *λ*_*1*_, throughout the run, and the travel into the state is kept at pre-lock-down levels. The testing rate *a* is set to 0%, representing no efforts to test the population. The model now presumes full mixing within the population.

Figures 1, 2, 3, S1 and S2 were prepared using MATLAB^42^, Wolfram Mathematica^43^ and final composite images were made using Adobe Animate^44^. Figures S3, S4, S5, S6 and S7 were prepared using R^37^, with the packages ggplot2^45^ and FME^16^.

## Supplementary Information

Supplementary Information.

## Data Availability

Code and related data are available https://anonymous.4open.science/r/covid-19_in_kerala-1060/.
