# Supplementary material for: Analysis of non-pharmaceutical interventions and their impacts on COVID-19 in Kerala
Source: Sci Rep. 2022 Jan 12;12:584. doi: 10.1038/s41598-021-04488-x (PMC8755744; doi:10.1038/s41598-021-04488-x)
Supplement: Supplementary file 1 — Supplementary Information. [file 41598_2021_4488_MOESM1_ESM.pdf]

Analysis of non-pharmaceutical interventions and their impacts on COVID-19 in Kerala

Supplementary Information

Elizabeth Goult[1,2], Shubha Sathyendranath[1*], Žarko Kovač[3], Christina Eunjin Kong[1], Petar Stipanović[3], Anas Abdulaziz[4], Nandini Menon[5], Grinson George[6] & Trevor Platt[1]

[1]Plymouth Marine Laboratory, Plymouth, UK

[2]Max Planck Institute for Infection Biology, Berlin, Germany

[3]Faculty of Science, University of Split, Split, Croatia

[4]CSIR National Institute of Oceanography, Kochi, India

[5]Nansen Environmental Research Centre – India, Kochi, India

[6]ICAR Central Marine Fisheries Research Institute, Kochi, India

[*]Correspondence to ssat@pml.ac.uk

Methods Table S1: Notation in the Kerala model

Notation	Definition	Unit
δ_E	Exposed people who travel into the state.	day^{-1}
δ_I	Infected people who travel into the state.	day^{-1}
δ_R	Recovered, immune people who travel into the state.	day^{-1}
δ_S	Susceptible people who travel into the state.	day^{-1}
δ_T	Total people travelling into the state.	day^{-1}
D	Deaths.	day^{-1}
E_h	Hospitalised exposed population.	-
E_o	Out-of-hospital exposed population.	-
H_h	Total hospitalised population.	-
H_o	Total out-of-hospital population.	-
I_h	Infected hospitalised population.	-
I_o	Infected out-of-hospital population.	-
R_h	Recovered hospitalised population.	-
R_o	Recovered out-of-hospital population.	-
S_h	Susceptible hospitalised population	-
S_o	Susceptible out-of-hospital population.	-

Table legend: Variables and definitions included in the Kerala COVID-19 model, and the model variants.

Methods Table S2: Parameters in the Kerala model.

Parameter	Definition	Value	Initial range	Unit
λ	Transmission rate.	Fitted	0-2	day^{-1}
μ_{se}	COVID-19 test sensitivity.	0.85[1]	-	-
μ_{sp}	COVID-19 test specificity.	1[1]	-	-
σ	Proportion of infected people who develop noticeable symptoms.	Fitted	0-1	day^{-1}
ω	Reciprocal of period from first negative test to release from hospital.	1[2]	-	day^{-1}
d	Probability of death for hospitalised infected people.	Fitted	0-1	day^{-1}
p	Rate at which exposed people become infectious.	1/5 [3]	-	day^{-1}
r	Recovery rate.	1/14[4]	-	day^{-1}
t_d	Delay in reporting of hospitalised cases.	7	-	days

Table legend: Parameters included in the Kerala COVID-19 model. Assigned parameter values from literature are given here. The ranges used for the initial MCMC simulation explorations are also detailed. Fitted parameters' values are displayed in Table S3.

Methods Table S3: Fitted parameter values

Parameter	Lowest SSR	Mean values	95% Credible interval	Inferred $\mathcal{R}_e$
λ_1	2.007	1.655	(0.9838, 2.014)	1.9
λ_2	0.1030	0.1583	(0.006852, 0.4229)	0.10
λ_3	2.377	1.851	(1.052, 2.413)	2.2
σ	0.9886	0.7804	(0.3625, 0.9926)	
d	0.0004854	0.0005075	(0.0003758, 0.0006842)	

Table legend: Parameter values and ranges. The values from the MCMC fitting with the lowest SSR (SSR = 858) are reported, along with the mean values (SSR = 859) and the 95% credible intervals of the parameters. The $\mathcal{R}_e$ value is calculated using the lowest SSR parameters. Note that the $\mathcal{R}_e$ value changes in the model with transmission rate, $\lambda_{i=1,2,3}$, but is also dependent on the values of r and σ.

Methods Table S4: Local sensitivity analysis.

Parameter	Sensitivity of total deaths	Sensitivity of total cases
λ_1	8.99	8.86
λ_2	0.32	0.44
λ_3	4.97	8.43
σ	-11.5	-14.5
d	0.996	6.48e-07
p	4.20	4.86
r	-1.46	-1.04
ω	-1.35e-06	-2.56e-06
μ_{sp}	-1.11e-07	-1.54e-07
μ_{se}	-5.47	-5.57

Table legend: Numerical estimates of the local sensitivity ($\frac{\partial y}{\partial \theta}.\frac{\Delta\theta}{\Delta y}$), where y is the variable of interest (total deaths and total cases modelled during the study period), and θ the parameter of interest (all parameters), implemented in the "sensFun" function from the R package FME[1]. The perturbation used was 1e-8, and the parameters and variables were not rescaled. A fully deterministic version of the model, where stochastic elements were set to their mean value, was used to remove the impact of stochastic variation on the sensitivity. The sensitivity of total deaths and of the total cases has modulus greater than one for $\lambda_1, \lambda_3, \sigma, p, r$ and μ_{se}.

Figure S1:

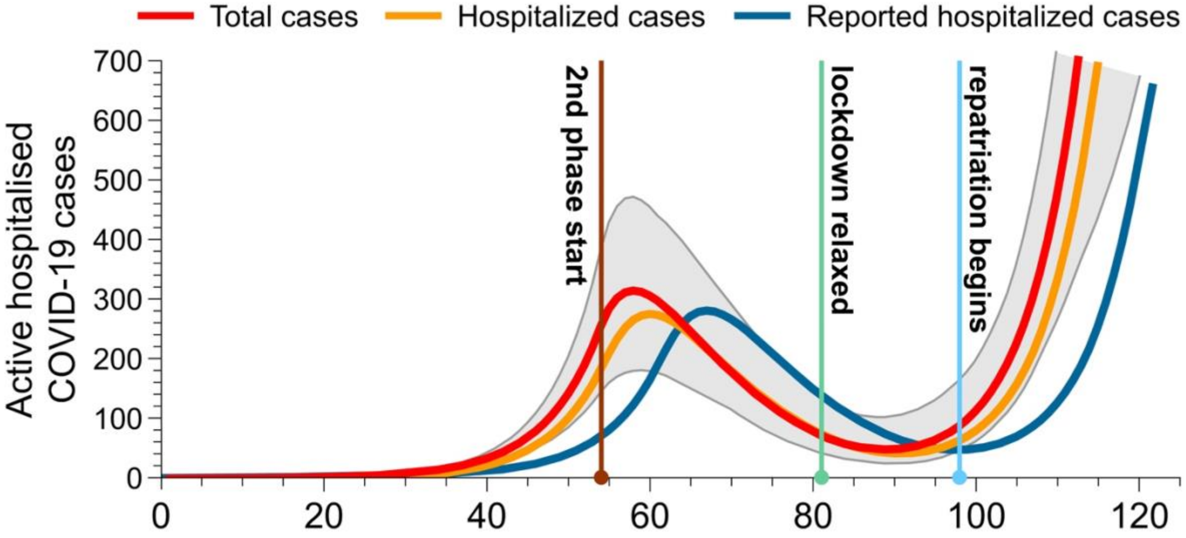

Figure legend: Comparison of total cases, hospitalisations and reported hospitalisations output by the model. The grey ribbon shows the minimum and maximum MCMC uncertainty in total cases. In the lowest SSR simulation the state had manged to contain all cases in hospitals, prior to day 88.

Figure S2:

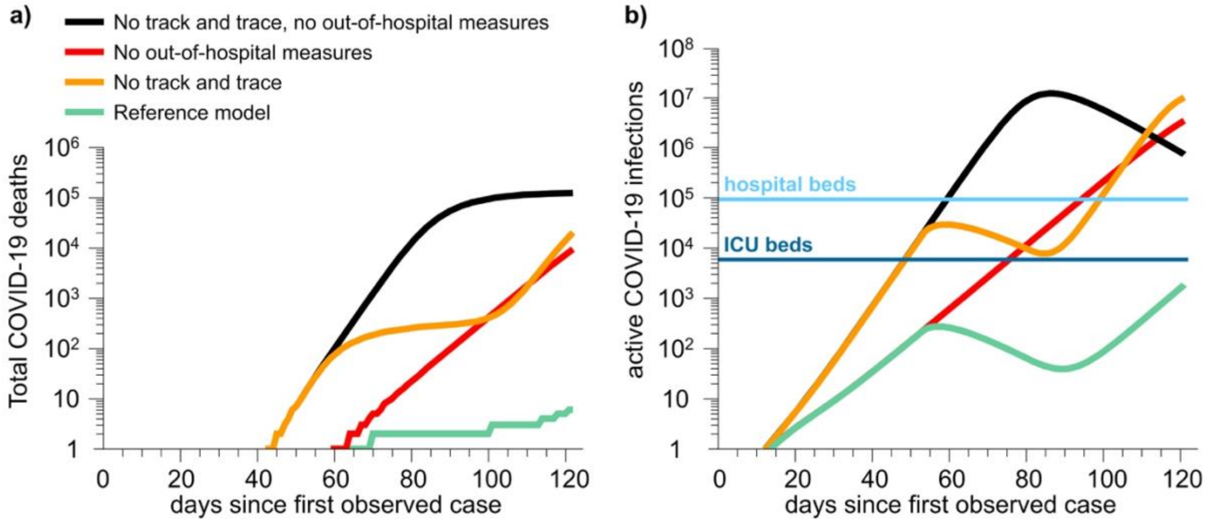

Figure legend: Comparing the model under different levels of action, with $\mathcal{R}_0$ set to 3 to reflect a scenario with no track and trace implemented. 95% confidence intervals are shown for the scenarios, calculated from the 100 repetitions to make up the aggregate mean.

Figure S3:

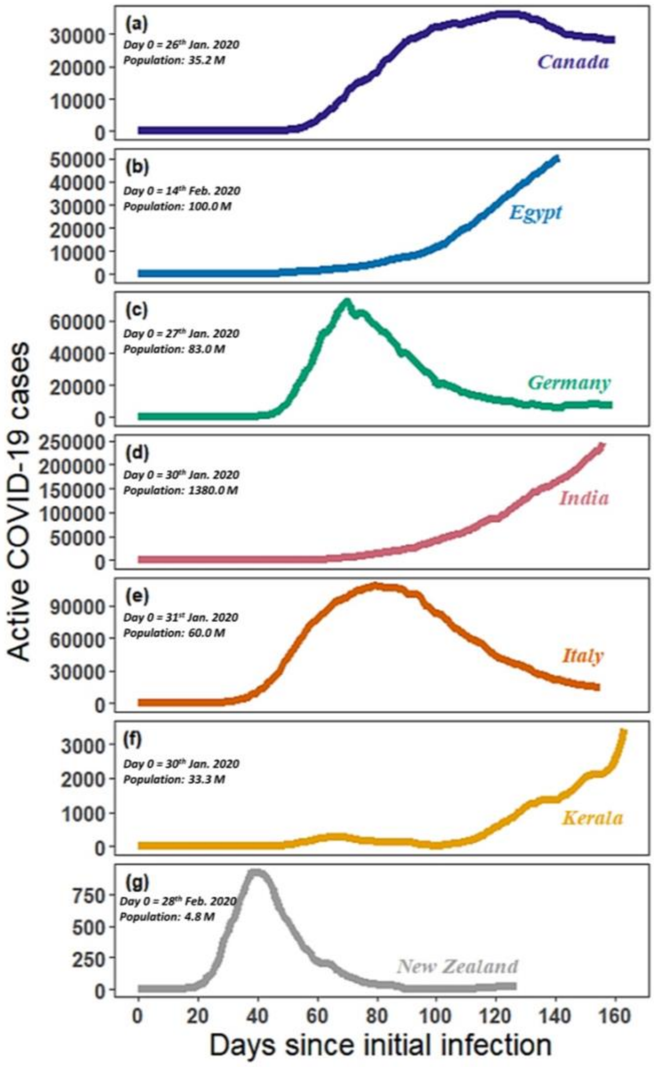

Figure legend: Comparing the Kerala timeseries of active cases of COVID-19 with selected countries.

Figure S4:

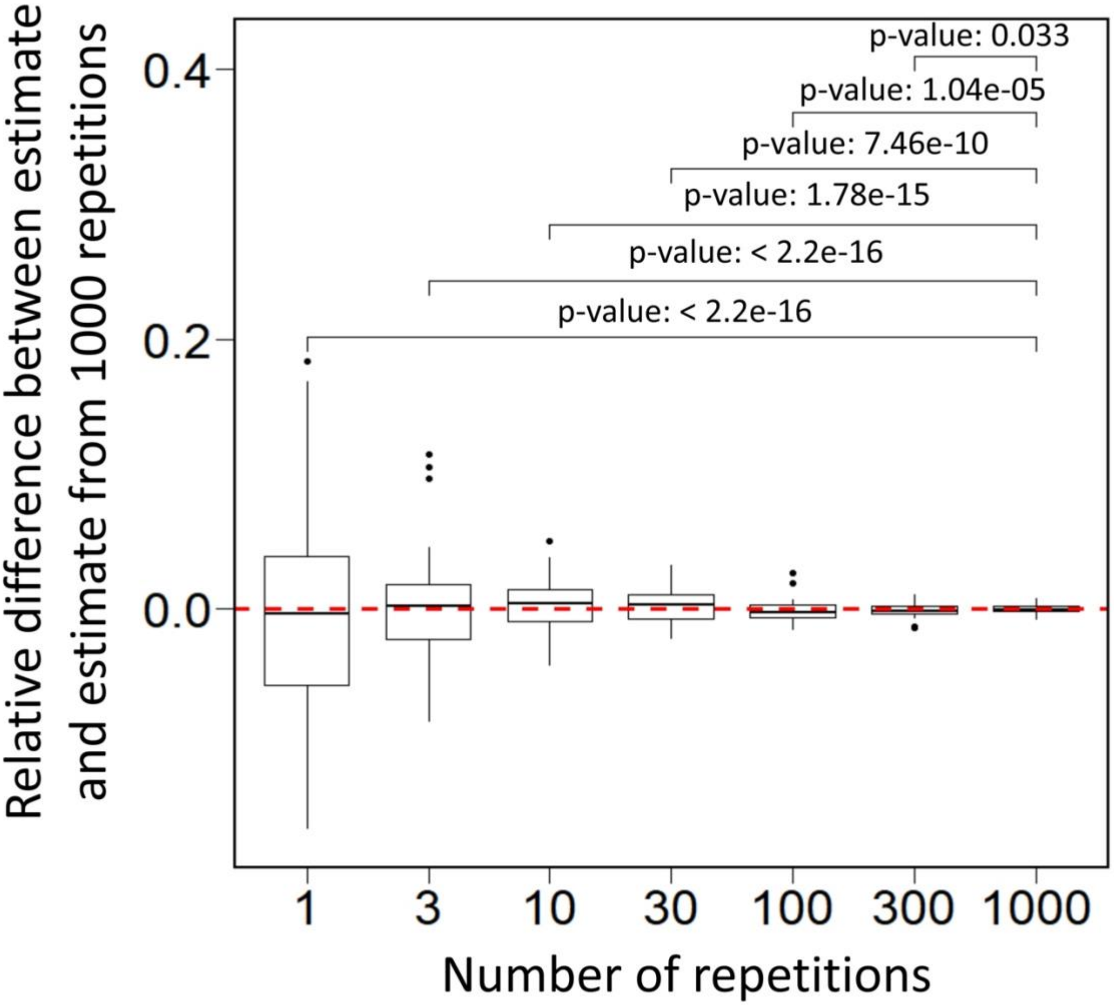

Figure legend: Sensitivity of results to the number of repetitions used to calculate the aggregate mean of the value. The aggregate values for the cumulative hospitalisations were calculated 30 times for each number of repetitions, then compared against the mean value for up to 1,000 repetitions. F-tests were then carried out between the sample variances and the variance for 1,000 repetitions to judge whether the number of repetitions were sufficient, with the p-values of these tests detailed in the plot. The red dashed line indicates 0 difference between the sample and the mean value for total cumulative hospitalisations for 1,000 repetitions.

Figure S5:

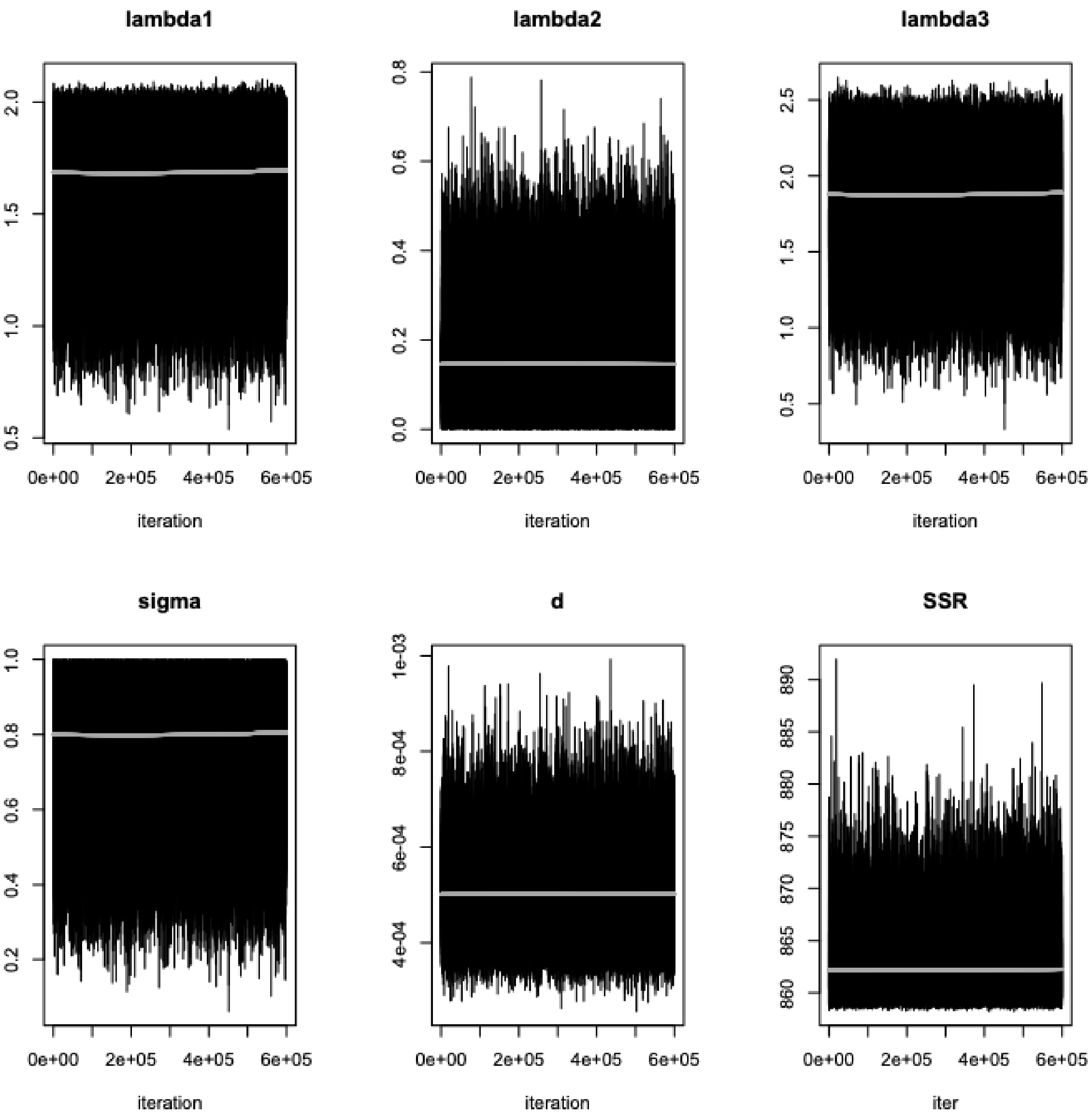

Figure legend: Parameters values by iteration resulting from the Monte Carlo Markov chain (MCMC) fitting. SSR measures the sum of weighted squared residuals per observed variable[5].

Figure S6:

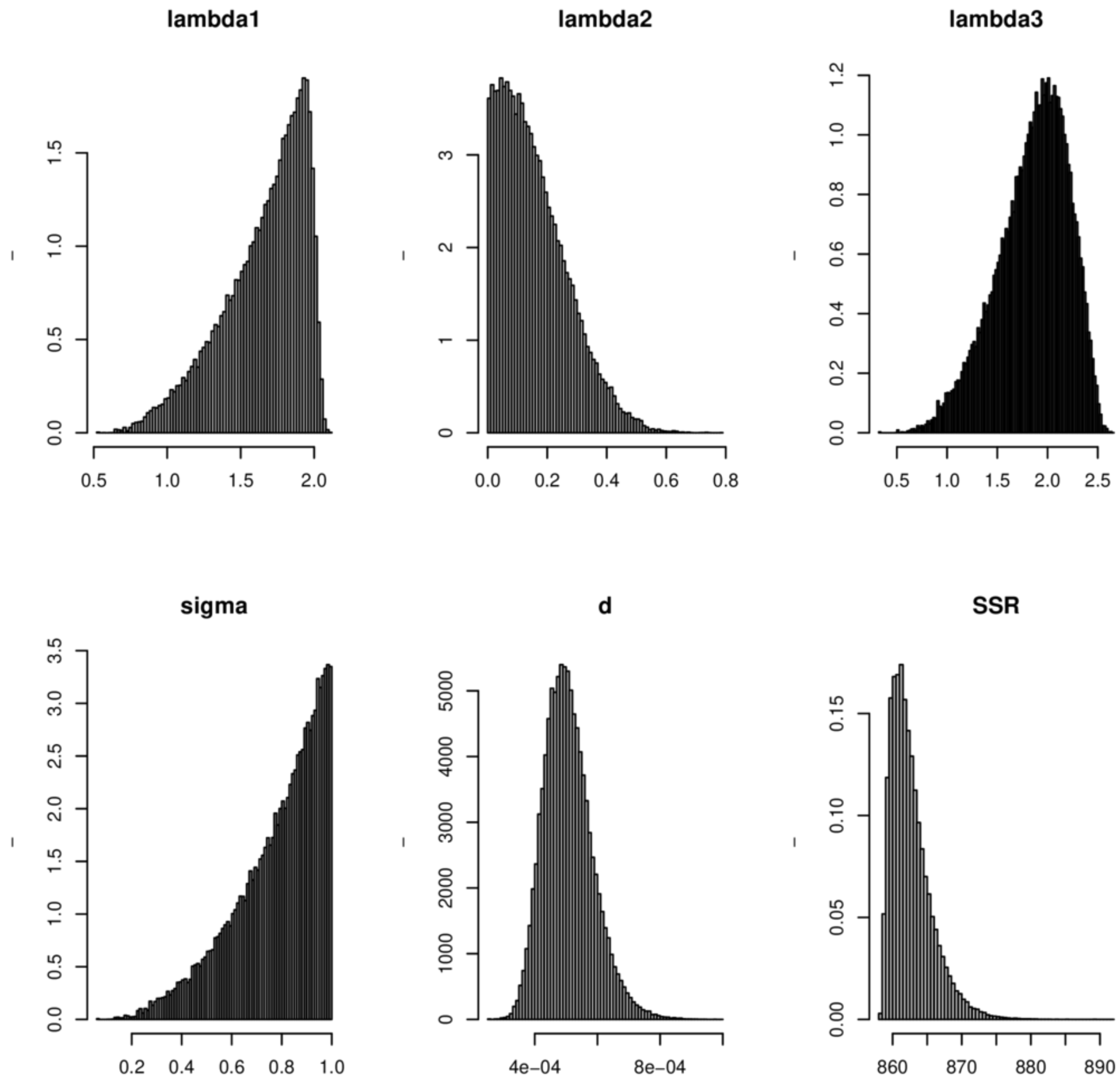

Figure legend: Parameters distributions resulting from the Monte Carlo Markov chain (MCMC) sampling.

Figure S7:

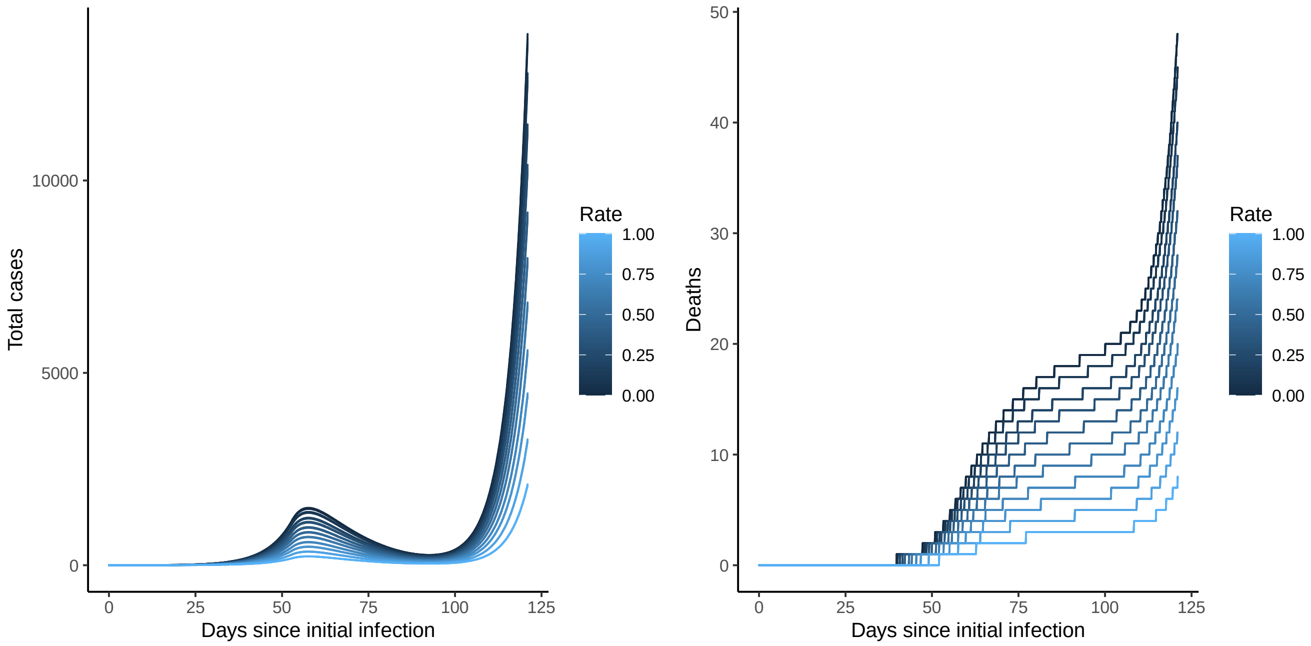

Figure legend: The impact of reduced testing rate for testing rates of 0%, 10%, 20%, 30%, 40%, 50%, 60%, 70%, 80%, 90% and 100%. The model was run with 300 repetitions for each testing rate, and the mean (dashed line) and 95% CI displayed. For a testing rate of 100% the model returns to the Kerala reference model (equations 1-8).

Likelihood function

The likelihood function for parameter set θ used by the adaptive Metropolis MCMC algorithm to fit the model to the data is defined as

$$L(\theta) = \sum_{j} L_j(\theta),$$

with $L_j(\theta) = -\sum_i log\left(g\left(x_{i,j}, \left(\widehat{x_{i,j}}\right)\right)\right)$.

Here $x_{i,j}$ is the ith observations of state j and $\widehat{x_{i,j}}$ is i[th] modelled value of state j. Hence,

$$g\left(x_{i,j}, \widehat{x_{i,j}}\right) = \begin{cases} f\left(\widehat{x_{i,j}}\right), f\left(\widehat{x_{i,j}}\right) \geq 0 \\ 0, otherwise. \end{cases}$$

Here, $f\left(\widehat{x_{i,j}}\right)$ is the normal distribution density function with mean $\mu = x_{i,j}$ and standard deviation equal to the standard deviation of x_j[5].

1. Grassly, N. *et al. Report 16: Role of testing in COVID-19 control.* 1-13. (Imperial College COVID-19 Response Team, 2020).

2. Government of Kerala. Daily Bulletin: COVID 19. *Directorate of Health Services.* https://dhs.kerala.gov.in/ (2020).

3. Lauer, S. A. *et al.* The Incubation Period of Coronavirus Disease 2019 (COVID-19) From Publicly Reported Confirmed Cases: Estimation and Application. *Ann Intern Med* **172**, 577-582 (2020).

4. Byrne, A.W. *et al.* Inferred duration of infectious period of SARS-CoV-2: rapid scoping review and analysis of available evidence for asymptomatic and symptomatic COVID-19 cases. *BMJ Open* **10,** e039856 (2020).

5. Soetaert K & Petzoldt T. Inverse Modelling, Sensitivity and Monte Carlo Analysis in R Using Package FME. *Journal of Statistical Software*, **33**(3), 1–28 (2010).
